# Epigenetic Control of Endocrine Pancreas Differentiation *in vitro*: Current Knowledge and Future Perspectives

**DOI:** 10.3389/fcell.2018.00141

**Published:** 2018-10-25

**Authors:** Veronica Astro, Antonio Adamo

**Affiliations:** Biological and Environmental Science and Engineering Division, KAUST Environmental Epigenetics Program, King Abdullah University of Science and Technology, Thuwal, Saudi Arabia

**Keywords:** β-cells, epigenetics, diabetes, *in vitro* differentiation, regenerative medicine

## Abstract

The raising worldwide prevalence of Type 1 and Type 2 diabetes mellitus (T1DM and T2DM) solicits the derivation of *in vitro* methods yielding mature and fully functional β-cells to be used in regenerative medicine. Several protocols to differentiate human embryonic stem cells (hESCs) and induced pluripotent stem cells (iPSCs) into human pancreatic β-like cells have recently been developed. These methods, coupled with a bioengineering approach using biocompatible encapsulating devices, have recently led to experimental clinical trials showing great promises to ultimately end the battle of diabetic patients for managing hyperglycemia. However, *in vitro* differentiation protocols face the challenge of achieving homogenous population of mono-hormonal insulin-secreting mature β-cells. Major epigenetic events such as DNA methylation, post-translational modification of histones and non-coding RNAs expression, orchestrate physiological endocrine pancreas specification into α-, β-, γ-, and δ-cells, both *in vivo* and *in vitro*. The dysregulation of such epigenetic processes is associated to multiple pancreatic disorders including diabetes. Understanding the epigenomic and transcriptomic landscape underlying endocrine pancreas development could, therefore, improve *in vitro* differentiation methods. In this review, we summarize the most effective protocols for *in vitro* differentiation of hESCs/hiPSCs toward pancreatic β-cells and we discuss the current limitations in the derivation of functional glucose-responsive, insulin-releasing β-cells. Moreover, we focus on the main transcriptional and epigenetic events leading to pancreatic specification and on the applicative potential of novel epigenetic drugs for the establishment of innovative pharmacological therapeutic approaches.

## Introduction

Insulin-dependent T1DM is characterized by selective autoimmune depletion of pancreatic β-cells resulting in deficient insulin release (Katsarou et al., 2017). Current pharmacological treatments mostly rely on the use of exogenous insulin to control glycemia (Pickup, 2012). The transplantation of cadaveric islets in combination with immunosuppressive therapies has been proven to reestablish normoglycemia up to several years but is limited by the scarcity of donor tissues (Bellin et al., 2012). Therefore, the availability of hESC-derived β-cells in combination with immune-protective devices could provide an innovative therapeutic approach for the treatment of the disease (Agulnick et al., 2015; Vegas et al., 2016). T2DM is a metabolic syndrome characterized by impaired glucose regulation resulting from insulin resistance and progressive loss of β-cells functionality. T2DM pharmacological protocols mainly target the endogenous response to glucose stimulation by increasing insulin sensitivity, insulin secretion, and glucose uptake (Marín-Peñalver et al., 2016). However, the efficacy of such treatments is progressively reduced due to eventual pancreatic β-cells failure (Kahn, 1994; Miller and Nguyen, 2014) and exogenous insulin administration is required. Thus, both T1DM and T2DM patients would benefit from regenerative medicine applications aimed at reconstituting physiological β-cells mass and function.

The adult human pancreas comprises two distinct endocrine and exocrine functional compartments. Acinar and ductal cells of the exocrine area occupy about 95% of the pancreas parenchyma, while islets of endocrine cells randomly scattered throughout the organ constitute the remaining 5% (Paris et al., 2004). These regions, known as the islets of Langerhans, contain a heterogeneous population of endocrine cells with specialized functions: α-, β-, γ-, δ-, and 𝜀-cells secreting glucagon (GCG), insulin (INS), pancreatic polypeptide (PPY), somatostatin (SST), and ghrelin (GHR) hormones, respectively. The breakthrough of single-cell RNA-Seq technology analysis has allowed the study of islet biology and its subpopulations at single cell level (Baron et al., 2016; Wang et al., 2016), thus opening the road to a deep understanding of pancreatic physiopathology at the molecular level. Moreover, the combination of techniques such as imaging mass spectrometry (IMS) (Jansson et al., 2016), mass cytometry (Spitzer and Nolan, 2016; Wang et al., 2016) and single cell RNA-Seq profiling of subpopulations of human and mouse islets (Muraro et al., 2016; Zeng et al., 2017), confirmed proteomic and transcriptomic signatures related to glucose sensing, uptake, and insulin releasing, but also revealed an unexpected heterogeneity in the transcriptomic pattern of individual β-cells and highlighted main molecular changes in the profiles of healthy and diabetic patients. Recent findings have identified up to five different β-cell subpopulations with diverging expression of subsets of genes implicated in insulin resistance, sensitivity, and diabetes [e.g., the adipokine RBP4, the free fatty acid receptor FFAR4 and the transcription factors (TFs) ID1 and ID3], oxidative metabolism and endoplasmic reticulum stress (Baron et al., 2016; Muraro et al., 2016; Segerstolpe et al., 2016). Despite these advances, further knowledge may come from a thorough analysis of the transcriptomic and proteomic signatures of different cells within the Langerhans islets and the generation of innovative organoid-based differentiation protocols mimicking the physiological three-dimensional (3D) architecture of pancreatic islets.

Human pluripotent stem cells (hPSCs) can serve as an inexhaustible source of differentiated cells due to their unique features of unlimited expansion and specification into virtually all somatic cells including pancreatic β-cells. However, to date, reproducing *in vitro* physiological conditions resulting in sustained β-cells maturation has been largely unsuccessful. Over the last decade, the increased understanding of the mechanisms regulating pancreatic differentiation has allowed the derivation of protocols to direct hPSCs toward insulin-producing β-like cells. While the available protocols efficiently yield differentiated intermediates, including definitive endoderm (DE), primitive gut tube-like (PGT), and pancreatic progenitors (PPs), they fail to generate functional, mono-hormonal β-cells. Consequently, the final stages of *in vitro* differentiation protocols result in a mixture of immature poly-hormonal endocrine cells featuring α-, β-, γ-, and δ-cells characteristics. Here, we dissect the temporally activated molecular events regulating pancreas specification *in vivo* and *in vitro* and discuss the role played by epigenetic modifiers in driving pancreas organogenesis and pathogenesis. Moreover, we explore the potential benefits of the implementation of drugs acting on epigenetic modifiers, also known as “epidrugs,” to optimize β-cells differentiation *in vitro* (Zullo et al., 2017).

## Differentiation of Hesc and Hipsc Into Pancreatic β-Cells: Closing the Gaps

Ontogenetic studies performed in rodents elucidated the sequential developmental steps that, following gastrulation and endodermal specification, give rise to pancreatic endocrine progenitors. The endodermal germinal layer undergoes an intricate patterning of invaginations leading to the formation of the PGT organized into foregut, midgut, and hindgut. The commitment of foregut tube endoderm cells toward PPs is driven by orchestrated signaling mediated by transforming growth factor-β (TGF-β) proteins of the activin/nodal families, bone morphogenic protein (BMP), retinoic acid (RA) and AKT/PI3K pathways (D’Amour et al., 2005; Gadue and Gordon, 2006). Formation of pre-pancreatic endoderm is subsequently marked by the induction of the pancreatic and duodenal homeobox 1 (PDX1) TF, a master regulator expressed by all common progenitors of the mature pancreas (Herrera et al., 2002). Subsequently, pancreagenesis progresses as PDX1+ precursors proliferate and sprout to form ventral and dorsal buddings, that in turn fuse to form a single organ. Induction of the TF NKX6.1 restricts PDX1+ cells fate to endocrine and ductal cells (Schaffer et al., 2010) and drives the expression of key β-cell specification genes (Pedersen et al., 2005; Wescott et al., 2009; Taylor et al., 2013; Shih et al., 2015). At this stage, subsequent waves of proliferation produce a dense epithelial bud, where Neurogenin-3 (NGN3) positive pre-endocrine cells emerge and differentiate into proto-islet structures expressing ISL LIM Homeobox 1 (ISL1), Neuronal Differentiation 1 (NEUROD1), and Insulinoma associated protein 1 (INSM1) (Rosa and German, 2004; Gierl, 2006; Jia et al., 2015). From these late progenitors, mono-hormonal INS+, GCG+, PPY+, SST+, and GHR+ cell subtypes arise. Leveraging this knowledge, several groups developed multistep *in vitro* protocols to generate mono-hormonal glucose-responsive and insulin-releasing β-cells from hPSCs (Rezania et al., 2014; Millman et al., 2016; Table 1). However, several technical and biological limitations have prevented the establishment of a robust and standardized differentiation protocol: first, differentiation efficiency varies across different hPSCs lines and might be affected by growth rate, cell density, and batch-to-batch inconsistency of Matrigel and serum derivatives (e.g., knockout serum replacement, and KSR) often used in *in vitro* methods (Blauwkamp et al., 2012; Zeng et al., 2016; Shi et al., 2017; Tchieu et al., 2017); second, the *in vivo* transplantation required for the final maturation of the *in vitro*-derived β-like cells is intrinsically linked to biological variability; third, the amount of insulin released and the response to glucose *stimuli* are not yet comparable to *bona fide* β-cells (e.g., cadaveric islets used as gold standard positive control), suggesting faults in glucose sensing and insulin secretion machinery (Pagliuca et al., 2014; Rezania et al., 2014).

**Table 1:** Reports describing the differentiation of hESC and hiPSC into pancreatic β-like cells.

			DE	PPs	Immature β-cells		β-like cells		
Reference	hESC/hiPSC lines	Differentiation conditions	DE markers (%)	PDX1+/NKX6.1+(%)	NKX6.1+/C-Peptide+ (%) or NKX6.1+/INS+ (%)	INS+/GCG+(%) or C-Peptide+/GCG+ (%)	*In vivo* Transplantation	Glucose homeostasis restoration	Glucose Stimulated Insulin Release (GSIS)
Pagliuca et al., 2014	HUES8hESC, hiPSCs	Spinning flasks	>95%SOX17+	n.t	NKX6.1+/C-Peptide+ 31–38%	C-Peptide +/GCG+ 8%	NOD-SCID and NRG-Akita mice	Yes	Yes (tested *in vitro* and *in vivo)*
Rezania et al., 2014	hESC HI	Adherent on Matrigel+ ALI	n.t	76%	NKX6.1+/INS+ 40–51%	INS+/GCG+ 21%	NOD-SCIDy (NSG) mice	Yes	Yes (tested *in vivo)*
Nostro et al., 2015	hESC HI, H9 MSC-, BJ-iPSC MEL-1, hiPSC38-2	Adherent on Matrigel	>93% CXCR4+/CD117+	n.q	negative	n.q	NSG mice	n.t	Yes (tested *in vivo)*
Millman et al., 2016	ND-1, ND-2;T1DM patient-derived hiPSCs	Spinning flasks and rotating plate	n.q	52–79%	NKX6.1+/C-Peptide+ 30%	n.q	NOD-SCID mice	Yes	Yes (tested *in vitro* and *in vivo)*
Russ et al., 2015	MEL-1	Orbital shaker	n.t	67–89%	NKX6.1+/INS+ 12%	C-Peptide +/GCG+ 13%	NSG mice	Yes	No (tested *in vitro)*
Yoshihara et al., 2016	HUVEC-derived hiPSC	Adherent on Matrigel	n.t	n.t.	NKX6.1+/INS+ 30%	INS+/GCG+ 15%	NOD-SCID mice	Yes (only using ERRy overexpressing cells)	Yes (tested *in vitro*, only upon ERRy overexpression)
Shi et al., 2017	hESC HI, HUES8	Adherent on Vitronectin+ ALI	56–86% CXCR4 +/SOX17 +	39–65%	NKX6.1+/C-Peptide+ 18%	C-Peptide +/GCG+ 10%	NOD-SCID mice	n.t	Yes (tested *in vitro* and *in vivo)*
Ghazizadeh et al., 2017	hESC HI, HUES8, hiPSCs	Adherent on Matrigel+ suspension or ALI from day 10-13	n.q	n.t	NKX6.1+/C-Peptide+ in ALI 18% in LA 9%	C-Peptide +/GCG+ in ALI 8% In LA 12%	NOD-SCID mice	Yes	Yes (tested *in vitro* and *in vivo)*
Agulnick et al., 2015	CyT49 hESC	Orbital shaker	n.t	75%	n.t	INS+/GCG+ 15% INS+/GCG-30%	NOD-SCID mice	n.t	Yes (tested *in vivo)*

*n.t, non-tested; n.q, not quantified; LA, low attachment; ALI, Air-liquid interphase; INS, Insulin; GCG, Glucagon; DE, Definitive Endoderm; PPs, Pancreatic Precursors. Columns 4–7 show the percentage of cells expressing stage-specific markers detected by FACS or immunofluorescence*.

During *in vitro* specification, three key stages can be considered informative check-points to predict the efficacy of the differentiation protocols:

### Specification of hPSCs Into DE and PGT

Induction of DE cells from hPSCs was initially achieved by combining Activin A (AA) and WNT3 recombinant proteins, activating the nodal/activin and β-catenin pathways, respectively (D’Amour et al., 2006). More recently, the glycogen synthase kinase 3β (GSK3-β) inhibitor and specific activator of the canonical Wnt/β-catenin pathway CHIR99021 was shown to induce DE more efficiently in combination with AA (Rezania et al., 2014). Lately, novel small molecules, including several ROCK inhibitors identified through a high-throughput screening approach, have further improved the differentiation of hPSCs toward DE and PPs (Korostylev et al., 2017). The molecular mechanism responsible for the increased formation of endoderm intermediates upon ROCK inhibition remains elusive, but similar results are observed using high density cultures (Toyoda et al., 2017) and 3D aggregation conditions (Takeuchi et al., 2014). This parallelism suggests that sustained reshaping of the cellular cytoskeleton could promote pancreatic specification *in vitro*. Consequently, an increased awareness that mechanical forces, cell–cell, and cell–substrate interactions boost pancreatic differentiation, encouraged scientists to implement pioneering expedients including rotating-bioreactors (Agulnick et al., 2015), microgravity chambers (Tanaka et al., 2013), hydrogel micro-well platforms (Bernard et al., 2012), and biomimetic scaffolds (Wang et al., 2017) which resulted in improved differentiation efficiency. However, current protocols yield about 85% of cells expressing the DE markers FOXA2, SOX17, and CXCR4 (Nostro et al., 2015; Zhu et al., 2016; Shi et al., 2017), but fail to produce fully homogenous populations of DE cells and require additional studies and optimization. According to several published protocols, further differentiation of DE cells into PGT can be achieved by the use of culture media containing fibroblast growth factor 10 (FGF10) or keratinocytes growth factor (KGF) (Kroon et al., 2008; Pagliuca et al., 2014). PGT specification leads to the formation of a compact epithelium expressing hepatocyte nuclear factors HNF4α, HNF1β, and HNF6 (Sampaziotis et al., 2017).

### Derivation of PDX1+ NKX6.1+ Pancreatic Progenitors

The derivation of PDX1+ progenitors from PGT cells is achieved by using RA in combination with BMP, sonic hedgehog (SHH) signaling inhibitors, and either FGF10 or KGF (Guo et al., 2013; Hua et al., 2013). Additionally, low concentrations of Protein kinase C (PKC) agonists such as (2S,5S)-(E,E)-8-(5-(4-(trifluoromethyl)phenyl)-2,4-pentadienoylamino)benzolactam (TPB) and Phorbol 12,13-dibutyrate (PDBu), further stimulate PDX1 induction (Rezania et al., 2014; Zhu et al., 2016). PDX1+ cells must progressively acquire expression of the master regulator NKX6.1 to further progress toward endocrine commitment into β-cells. This is achieved by the use of endothelial growth factor (EGF), nicotinamide and Noggin (Nostro et al., 2015). It has been proposed that a subtle and short-term stimulation of BMP and SHH signaling is crucial to commit toward a mono-hormonal endocrine fate characterized by the expression of NGN3 in PDX1+/NKX6.1+ precursors, from which NKX2.2+ and subsequently C-Peptide+/INS+ cells emerge (Jennings et al., 2013; Russ et al., 2015). Conversely, extended stimulation promotes NGN3 expression in a subpopulation of PDX1+/NKX6.1- cells and lead to C-Peptide+/GCG+ poly-hormonal cells (Gu et al., 2002; Nostro et al., 2015; Russ et al., 2015).

### Specification of PPs Into β-Like Cells

Existing *in vitro* protocols promote the generation of immature poly-hormonal cells (e.g., GCG+/INS+; SST+/INS+; and PPY+/INS+) characterized by defective glucose-sensing insulin-releasing characteristics (Table 1; D’Amour et al., 2006; Kroon et al., 2008; Rezania et al., 2012). The immature state of the *in vitro*-derived β-like cells recapitulates features of the immature fetal pancreas, whose functional maturation occurs postnatally during the transition from weaning to increased carbohydrate intake, and is likely triggered by the transition from glycolysis to oxidative phosphorylation (Aguayo-Mazzucato et al., 2013; Stolovich-Rain et al., 2015). In line with these observations, the exogenous expression of a transcriptional regulator of mitochondrial oxidative metabolism such as the estrogen-related receptor gamma (ERRγ) ameliorates *in vitro* maturation and glucose stimulated insulin secretion (GSIS) of iPSC-derived β-like cells (Yoshihara et al., 2016). Consistently, an independent study demonstrated that cellular homeostasis and metabolism are influenced by oxygen tension and that hyperoxic cell culture conditions improve differentiation toward endocrine β-cells (Hakim et al., 2014). While the optimal *in vitro* culture conditions to further specify poly-hormonal cells into single-signature β-cells remain elusive, xenotransplantation of these cells into diabetic mice is sufficient to drive the final maturation into INS+ cells expressing specific *bona fide* markers of β-cells and to reestablish normoglycemia (Pagliuca et al., 2014; Rezania et al., 2014; Dhawan et al., 2015; Russ et al., 2015; Millman et al., 2016). The encouraging data obtained in diabetic mice and the generation of biocompatible and immune-protective devices have paved the way to approve clinical trials for T1DM patients (ViaCyte Inc., Clinical trial number: NCT03162926). The main goal of these trials is to assess the safety, tolerability, and efficacy of the treatment related to tumorigenic risk, scarce immune-tolerance, and inefficient vascularization.

## Epigenetics and *in vitro* Differentiation

Seminal work from Sander’s group thoroughly characterized the epigenetic landscape of endodermal progenitors and pancreatic β-cells precursors derived from hESCs (Xie et al., 2013). During endodermal differentiation, hPSCs undergo dynamic epigenetic remodeling processes regulated by Polycomb group proteins (PcG) and the histone H3 lysine 27 (H3K27) demethylase KDM6B. Specifically, differentiation of hPSCs into DE is accompanied by the resolution of bivalent domains associated with critical DE marker genes such as SOX17, EOMES, and GATA6 whose induction is KDM6B-dependent (Xie et al., 2013). As differentiation toward later stages of pancreatic specification proceeds, DE markers become silenced in a PcG-dependent manner and late markers are progressively induced. Later work from the same laboratory demonstrated that the enhancers associated with lineage-specific TFs that regulate pancreas development (including PDX1, SOX9, and PROX1) are in a “poised” state (H3K4me1 only) at the PGT stage. This process primes the PGT progenitors to respond to subsequent differentiation cues and is regulated by the TFs FOXA1 and FOXA2 (Wang et al., 2015). Subsequently, lineage-specific and H3K4me1-marked enhancers are terminally activated by acquiring H3K27 acetylation (H3K4me1/H3K27ac) (Wang et al., 2015). Over the last decade, several groups have tested multiple epidrugs to optimize β-cell differentiation *in vitro* (Table 2). In 2009, Melton’s group identified two compounds, IDE1 and IDE2, from a library of HDACs inhibitors, that drive efficient differentiation of mouse ESCs into DE through induction of the endodermal marker Sox17 (Borowiak et al., 2009). The class-I HDAC inhibitors sodium butyrate and valproic acid have also been used in combination with AA to prime hESCs toward DE (Hay et al., 2008; Kondo et al., 2014), while the demethylating agent 5^′^-Azadeoxycytidine (AZA) has been shown to stimulate NGN3 expression (Lefebvre et al., 2010). Thus, the increased knowledge on the epigenetic and transcriptional landscape of PPs and mature β-cells coupled to a systematic high-throughput screening of epidrugs at each step of differentiation offers new opportunities for the optimization of the current protocols.

**Table 2:** Epigenetic modulators implemented in protocols for *in vitro* differentiation into β-like cells.

Categories	Compound Name	Mechanism of action	References
HDAC Inhibitors	IDE1 and IDE2	Induces SOX17 and FOXA2 expression at DE stage	Borowiak et al., 2009
	Sodium Butyrate	Induces the expression of DE markers in cooperation with AA	Hay et al., 2008
	Valproic acid	Ameliorates the differentiation toward endoderm lineages	Kondo et al., 2014
DNA methylation inhibitors	5-Aza-2-deoxycytidine (5-Aza-DC)	Favors NGN3 expression by inhibiting the DNA methyltransferase (Dnmt)	Lefebvre et al., 2010
		Increases PDX1 expression by inhibiting Dnmt	Manzar et al., 2017
Histone marks modulators	Nicotinamide	Promotes SIRT1-mediated histone demethylase acetylation and increases NKX6.1 expression	Nostro et al., 2015; Luo et al., 2016
	BRD7552	Modulates histone H3 tail modifications that induce PDX1 expression	Yuan et al., 2013

## Transcriptional and Epigenetic (Mis)Regulation of the Endocrine Pancreas

Environmental variables such as lifestyle, diet, and active exercise impact the overall health status of individuals through complex homeostatic mechanisms that involve transcriptional and epigenetic remodeling (Ling and Groop, 2009; Jerram et al., 2017). These variables could be particularly relevant in concomitance with genetic predispositions including haploinsufficiency of key genes regulating pancreatic development and function (Seidman and Seidman, 2002). For example, heterozygous loss of function (LoF) mutations of the pancreatic TFs HNF1α, HNF4α and HNF1β are associated to the maturity-onset diabetes of the young (MODY) (Ryffel, 2001), and the wide spectrum of phenotypes associated to these mutations could result from the integration with non-genetic variables. Work from Huangfu’s laboratory has recently dissected the role played by the pancreatic TFs GATA4 and GATA6 during stepwise differentiation of hESCs toward β-cells. They demonstrated that GATA6 expression is crucial to convert hESCs into DE, while GATA4 is essential for the specification of DE-PGT into PPs through a mechanism not reproducible in *in vivo* studies performed on mouse models (Shi et al., 2017).

Several reports have highlighted the pivotal role played by key epigenetic modifiers such as Dnmt1, PRC1/PRC2, and MLLs complexes during pancreatic development and how their dysregulation may lead to diabetes progression. For instance, β-cell specific Dnmt3A-KO mice show aberrant expression of major developmental metabolic genes, such as herokinase 1 (hk1) and lactate dehydrogenase A (ldhA), resulting in defective GSIS in the postnatal life (Dhawan et al., 2015). The DNA methylation state of promoters of genes regulating pancreatic specification such as INS, is also important for regulating β-cell mass and functions during aging (Avrahami et al., 2015). Dhawan et al. demonstrated that β-cells-specific DNA methyltransferase Dnmt1 inactivation leads to loss of β-cell mass and concomitant *trans*-differentiation into α-cells (Dhawan et al., 2011). This plasticity is mediated by the reactivation of the α-cell-restricted TF Arx and simultaneous repression of the β-cell-restricted TF Pax4. These data are coherent with a symmetrical study by Collombat et al. (2009) in which the overexpression of Pax4 drives conversion of α-cells into β-cells (Collombat et al., 2009). Moreover, Talchai et al. showed that β-cell-specific Foxo1 deletion in mice leads to reduced β-cell mass due to induced β-cells dedifferentiation and reactivation of TFs markers of endocrine progenitors (Talchai et al., 2012). More evidence demonstrated that the histone acetyltransferase (HAT) p300 and the histone methyltransferase (HMT) Set9 are responsible for the glucose-dependent insulin gene activation (Andrali et al., 2008). Moreover, combining immunostaining techniques, deep epigenome mapping, and single-cell transcriptomics Lu and colleagues identified shared aberrant chromatin signatures in T2DM patients and diabetic mouse models (Lu et al., 2018). Reduced levels of mature β-cells markers such as PDX1, MAFA, NKX6.1, NKX2.2, and UCN3, are accompanied by a marked H3K27me3 nuclear signal depletion in diabetic versus control islets, a change linked to the loss of PCR2 functions. Analyzing islets obtained from a β-cell restricted KO mouse strain for the embryonic ectoderm development PRC2 subunit (Eed-KO mice), the authors demonstrated a simultaneous shutdown of mature β-cells TFs (Neurod1, NKX6.1, and Mafa), the upregulation of earlier PP markers (i.e., Gata6, Gata4, Onecut2, and Foxa1) and increased levels of the dedifferentiation marker Gli2. These data indicate that PRC2 maintains global silencing in terminally differentiated β-cells and that PCR2-insufficiency contributes to the onset of T2DM (Lu et al., 2018). The transcriptomic and epigenomic analysis of healthy and T2DM pancreatic tissues has recently identified epigenomic and transcriptomic signatures associated to diabetes including impaired distribution of DNA methylation and histone modification marks at key genes regulating β-pancreatic cell maturation (Dayeh et al., 2014; Olsson et al., 2014; Rui et al., 2016). Overall, the accumulated evidence suggests that active epigenetic remodeling is occurring during progression of T2DM and that epigenetic memory-related mechanisms may influence the outcome of the pharmacological treatment to restore normo-glycemia.

## Concluding Remarks and Future Perspectives

The increasing prevalence of T1DM and T2DM demands derivation of novel regenerative medicine approaches aimed at reconstituting functional β-cells. Before the ground-breaking invention of the somatic cell reprogramming technique (Takahashi and Yamanaka, 2006; Takahashi et al., 2007), the study of diabetes largely relied on the use of bioptic tissues obtained from post-mortem healthy or diabetic pancreatic tissues and mouse models. The availability of hESC and hiPSC overcame these limitations allowing the study of the onset and progression of diseases “in a dish” and providing an unprecedented cellular *in vitro* drug discovery platform (Tiscornia et al., 2011; Soldner and Jaenisch, 2012). The derivation of diabetic hPSC-derived β-cells offers a novel tool for the study of the transcriptional and epigenetic dysregulation resulting from the integration of genetic backgrounds and environmental variables. In fact, aberrant deposition of epigenetic marks is a well-known molecular event associated to diabetes progression and epigenetic memory-related mechanisms may influence the outcome of the pharmacological treatments to restore normo-glycemia. For example, T1DM has been linked to dysregulated epigenetic signatures at critical genes involved in self-immunity and regulation of glucose homeostasis (Jayaraman et al., 2013; Zullo et al., 2017) Notably, inhibitors targeting both HATs and histone deacetylases (HDACs) are currently used in T2DM clinical trials (Sommese et al., 2017) and Class-II HDACs inhibitors are particularly promising since they simultaneously exert anti-inflammatory effect, amelioration of insulin sensitivity and enhanced β-cells maturation. The availability of next generation sequencing techniques coupled with single-cell multi-omics analysis offers the unprecedented possibility to obtain a comprehensive understanding of the epigenetic and transcriptional mechanisms regulating pancreagenesis. This could lead to personalized epidrugs–based pharmacological treatments and to improved culture conditions for the production of hPSC-derived β-cells *in vitro*. Finally, increased epigenetic knowledge would allow exploration of alternative therapeutic approaches such as the conversion of fibroblasts and α-cells to-β cells, a process that can be promoted by the use of 5-AZA-DC (Table 2; Lefebvre et al., 2010; Katz et al., 2013; Pennarossa and Brevini, 2013).

## Author Contributions

VA and AA conceived the work and wrote the manuscript.

## Conflict of Interest Statement

The authors declare that the research was conducted in the absence of any commercial or financial relationships that could be construed as a potential conflict of interest.
